# Lipid droplets fuel SARS-CoV-2 replication and production of inflammatory mediators

**DOI:** 10.1371/journal.ppat.1009127

**Published:** 2020-12-16

**Authors:** Suelen Silva Gomes Dias, Vinicius Cardoso Soares, André C. Ferreira, Carolina Q. Sacramento, Natalia Fintelman-Rodrigues, Jairo R. Temerozo, Lívia Teixeira, Marcos Alexandre Nunes da Silva, Ester Barreto, Mayara Mattos, Caroline S. de Freitas, Isaclaudia G. Azevedo-Quintanilha, Pedro Paulo A. Manso, Milene D. Miranda, Marilda Mendonça Siqueira, Eugenio D. Hottz, Camila R. R. Pão, Dumith C. Bou-Habib, Debora F. Barreto-Vieira, Fernando A. Bozza, Thiago M. L. Souza, Patricia T. Bozza

**Affiliations:** 1 Laboratório de Imunofarmacologia, Instituto Oswaldo Cruz (IOC), Fundação Oswaldo Cruz (FIOCRUZ), Rio de Janeiro, Rio de Janeiro, Brazil; 2 Programa de Imunologia e Inflamação, Universidade Federal do Rio de Janeiro, UFRJ, Rio de Janeiro, Rio de Janeiro, Brazil; 3 Centro De Desenvolvimento Tecnológico Em Saúde (CDTS) and National Institute for Science and Technology on Innovation on Diseases of Neglected Populations (INCT/IDNP), FIOCRUZ, Rio de Janeiro, Brasil; 4 Universidade Iguaçu, Nova Iguaçu, Rio de Janeiro, Brazil; 5 Laboratório de Pesquisas sobre o Timo and Instituto National de Ciencia e Tecnologia em Neuroimunomodulação (INCT/NIM), Instituto Oswaldo Cruz (FIOCRUZ), Rio de Janeiro, Rio de Janeiro, Brazil; 6 Laboratório de Morfologia e Morfogênese Viral, Instituto Oswaldo Cruz (IOC), Fundação Oswaldo Cruz (FIOCRUZ), Rio de Janeiro, Rio de Janeiro, Brazil; 7 Laboratorio de Patologia, Instituto Oswaldo Cruz (IOC), Fundação Oswaldo Cruz (FIOCRUZ), Rio de Janeiro, Rio de Janeiro, Brazil; 8 Laboratório de Vírus Respiratórios e do Sarampo, Instituto Oswaldo Cruz (FIOCRUZ), Rio de Janeiro, Rio de Janeiro, Brazil; 9 Laboratorio de Imunotrombose, Departamento de Bioquímica, Universidade Federal de Juiz de Fora, Juiz de Fora, Minas Gerais, Brazil; 10 Instituto Nacional de Infectologia Evandro Chagas (INI), FIOCRUZ, Rio de Janeiro, Brazil; 11 Instituto D’Or de Pesquisa e Ensino (IDOR), Rio de Janeiro, Brazil; Erasmus Medical Center, NETHERLANDS

## Abstract

Viruses are obligate intracellular parasites that make use of the host metabolic machineries to meet their biosynthetic needs. Thus, identifying the host pathways essential for the virus replication may lead to potential targets for therapeutic intervention. The mechanisms and pathways explored by SARS-CoV-2 to support its replication within host cells are not fully known. Lipid droplets (LD) are organelles with major functions in lipid metabolism, energy homeostasis and intracellular transport, and have multiple roles in infections and inflammation. Here we described that monocytes from COVID-19 patients have an increased LD accumulation compared to SARS-CoV-2 negative donors. *In vitro*, SARS-CoV-2 infection were seen to modulate pathways of lipid synthesis and uptake as monitored by testing for CD36, SREBP-1, PPARγ, and DGAT-1 expression in monocytes and triggered LD formation in different human cell lines. LDs were found in close apposition with SARS-CoV-2 proteins and double-stranded (ds)-RNA in infected Vero cells. Electron microscopy (EM) analysis of SARS-CoV-2 infected Vero cells show viral particles colocalizing with LDs, suggestive that LDs might serve as an assembly platform. Pharmacological modulation of LD formation by inhibition of DGAT-1 with A922500 significantly inhibited SARS-CoV-2 replication as well as reduced production of mediators pro-inflammatory response. Taken together, we demonstrate the essential role of lipid metabolic reprograming and LD formation in SARS-CoV-2 replication and pathogenesis, opening new opportunities for therapeutic strategies to COVID-19.

## Author summary

In spite of the enormous scientific efforts to understand mechanisms of SARS-CoV2-induced disease and to develop strategies to control COVID-19 pandemic, many aspects of SARS-CoV2 biology and pathogenesis remain elusive. Several RNA viruses are able to modulate the host lipid metabolism and to recruit LDs to enhance their replication/particle assembling capacity through mechanisms that vary according to the virus and the host cell infected. The mechanisms and pathways explored by SARS-CoV-2 to support its replication within host cells are still largely unknown. Here we demonstrated that lipid droplets (LDs) participate in SARS-CoV2 infection favoring virus replication and heightening inflammatory mediator production. SARS-CoV2 infection increased the expression of key proteins in the regulation of lipid metabolism and the amounts of LDs per cell. In addition, we have found SARS-CoV2 and/or its components associated with LDs in infected cells, suggestive that LDs are recruited as part of replication compartment. Moreover, pharmacological inhibition of DGAT-1, a key enzyme for LD formation, reduces SARS-CoV2 replication, inflammatory mediator production and cell death. Our findings contribute to unveil the complex mechanism by which SARS-CoV-2 make use of cellular metabolism and organelles to coordinate different steps of the viral replication cycle and host immunity, opening new perspectives for SARS-CoV2 antiviral development.

## Introduction

The coronavirus disease 2019 (COVID-19) caused by the novel severe acute respiratory syndrome-coronavirus 2 (SARS-CoV-2) has rapidly spread in a pandemic, representing an unprecedented health, social and economic threat worldwide [1,2]. This newly emerged SARS-CoV-2 belongs to the *Betacoronavirus* genus of the subfamily *Orthocoronavirinae* in the *Coronaviridae* family. Like other Coronavirus, the SARS-CoV-2 is an enveloped non-segmented positive-sense RNA (+RNA) virus [3], which genome sequence is similar to the already known SARS-CoV [4,5]. Despite the similarity with other members of the *Betacoronavirus* genus, the pathogenesis of SARS-CoV-2 infection presents unique properties that contribute to its severity and pandemic-scale spread. Therefore, it is necessary to understand how the virus interacts and manipulate host cell metabolism to develop novel strategies to control the clinical progression of the infection and to limit the SARS-CoV-2 pandemic.

Viruses are obligated intracellular parasites that require host cell machinery to replicate [6–8]. Viruses interact with several intracellular structures and have the ability to reprogram the cellular metabolism to benefit viral replication [9–11]. Increasing evidence points at major roles of lipid droplets (LD) for virus replication cycle and pathogenesis, highlighting the potential of these organelles as targets for drug development. Many studies have demonstrated the interaction of viral molecules with LD-related components, and the relevance of these organelles for viral replication as already demonstrated in several positive-strand RNA (+ RNA) viruses such as *Flaviviridae* members, rotavirus and reovirus [12–18]. Accordingly, pharmacological interventions that inhibit enzymes associated with lipid metabolism, as in fatty acid synthesis and esterification, impact the formation of LDs which in turn reduce viral replication and assembly [10,12,15,19–22]. In addition, LDs play an important role in the infection pathogenesis and inflammatory processes [23,24].

Here we demonstrate major effects of SARS-CoV-2 to modulate cellular lipid metabolism in human cells favoring increased de novo lipid synthesis and lipid remodeling, leading to increased LD accumulation in human cells. We also reported increased LD accumulation in monocytes from COVID-19 patients when compared to healthy volunteers. Inhibition of LD biogenesis with a pharmacological inhibitor of acyl-CoA:diacylglycerol acyltransferase-1 (DGAT-1) blocks viral replication, pro-inflammatory mediator production and prevented cell death. Collectively, our results uncover details of viral manipulation of host cell lipid metabolism to allow SARS-CoV-2 replication and may provide new insights for antiviral therapies.

## Results

### SARS-CoV-2 infection upregulates lipid metabolism, increasing LD biogenesis in human cells

Viruses have the ability to modulate cellular metabolism with benefits for their own replication. Several +RNA viruses, including members of *Flaviviridae* family, as HCV [13,25,26], DENV [17,27], as well as reovirus [16], norovirus [28] and poliovirus [29] modify the lipid metabolism in different cells and trigger LD formation, using these host organelles at different steps of their replicative cycle.

**Fig 1:**
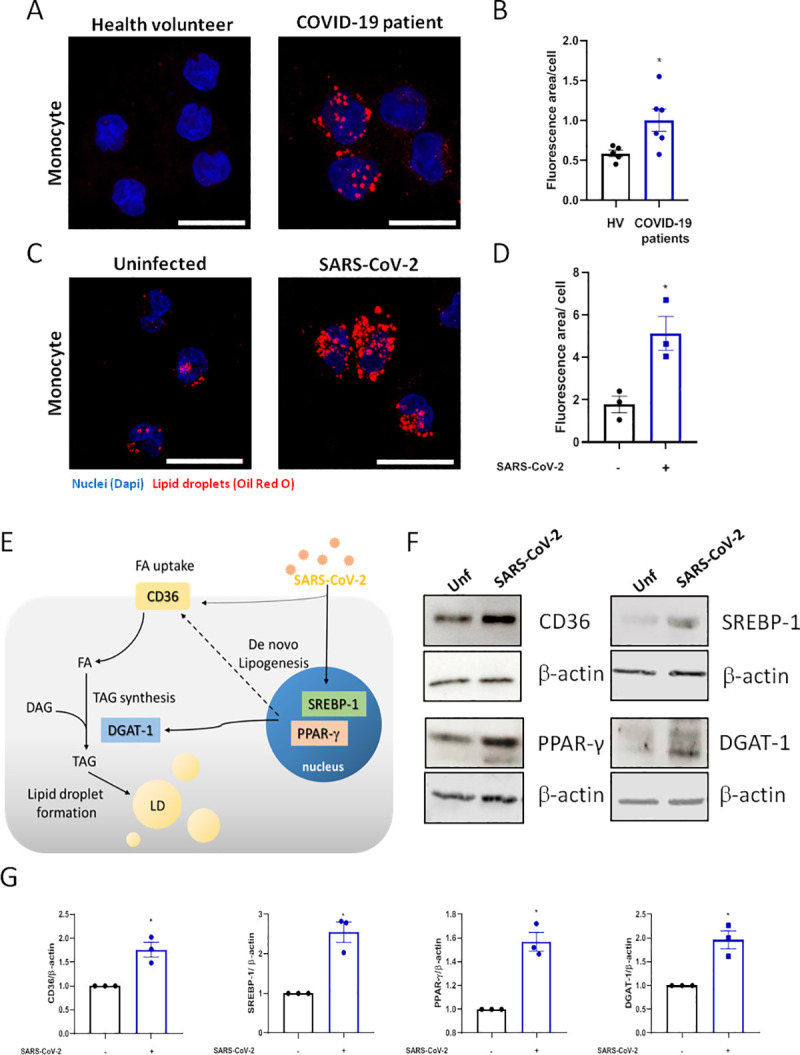
SARS-CoV-2 infection modulates the lipid metabolism in human monocytes. (A and C) LDs were captured by fluorescent microscopy after Oil Red O staining (Red) and nuclei stained with DAPI (Blue). (A) Representative images of monocytes from COVID-19 patients and health volunteers. (C) Representative images of human monocytes obtained from PBMC infected by SARS-CoV-2 with MOI of 0.01 for 24 hours. Scale bar 20μm. (B and D) LDs were evaluated by ImageJ software analysis by the measurement of the fluorescent area. (E) Representative scheme of the increase of proteins associated with lipid metabolism by SARS-CoV-2 infection in monocyte can regulate the lipid droplet formation. (F) Monocytes were infected by SARS-CoV-2 with MOI of 0.01 during 24h. Cell lysates were collected for the detection of CD36, PPAR-γ, SREBP-1, DGAT-1 by Western blotting. β-actin levels were used for control of protein loading. (G) Densitometric evaluation of data of panel 1F. Data are expressed as mean ± SEM of five healthy volunteers (HV) and six COVID-19 patients for ex vivo experiments and three healthy donors for LDs staining and western blot. *p < 0.05 versus health volunteers or uninfected cells.

Here, we demonstrated increased LD accumulation in human monocytes from COVID-19 patients when compared with healthy volunteers (Fig 1A and 1B). Likewise, we demonstrated that in vitro infection of cells and cell lines with SARS-CoV-2 with a multiplicity of infection (MOI) of 0.01 triggers the increase of LDs in primary human monocytes within 24 hours (Fig 1C and 1D), as well as in a human lung epithelial cell line (A549) (S1A Fig), and human lung microvascular endothelial cell line (HMVEC-L) after 48 hours post-infection (S1B Fig).

Lipid metabolism alterations in cells and plasma are emerging as major phenotypes during COVID-19 and SARS-CoV-2 infection [30]. To gain insights into the mechanisms involved in LD formation, we evaluated whether SARS-CoV-2 infection could modulate the expression of the proteins associated with lipid metabolism involved in lipid uptake and de novo lipid synthesis (Fig 1E). As shown in Fig 1E, 1F and 1G, SARS-CoV-2 infection of human primary monocytes up-regulated the pathways involved in lipid uptake such as CD36, the major transcriptional factors involved in lipogenesis, PPARγ and SREBP-1, and the enzyme DGAT-1, which is involved in triacylglycerol synthesis, after 24 hours of infection.

Altogether, these data suggest that SARS-CoV-2 is able to modulate multiple pathways of lipid metabolism, including in immune cells from COVID-19 patients, culminating in new LD assembling in human cells.

### Inhibition of LD formation decreases viral replication and prevents cell death in SARS-CoV-2 infected monocytes

**Fig 2:**
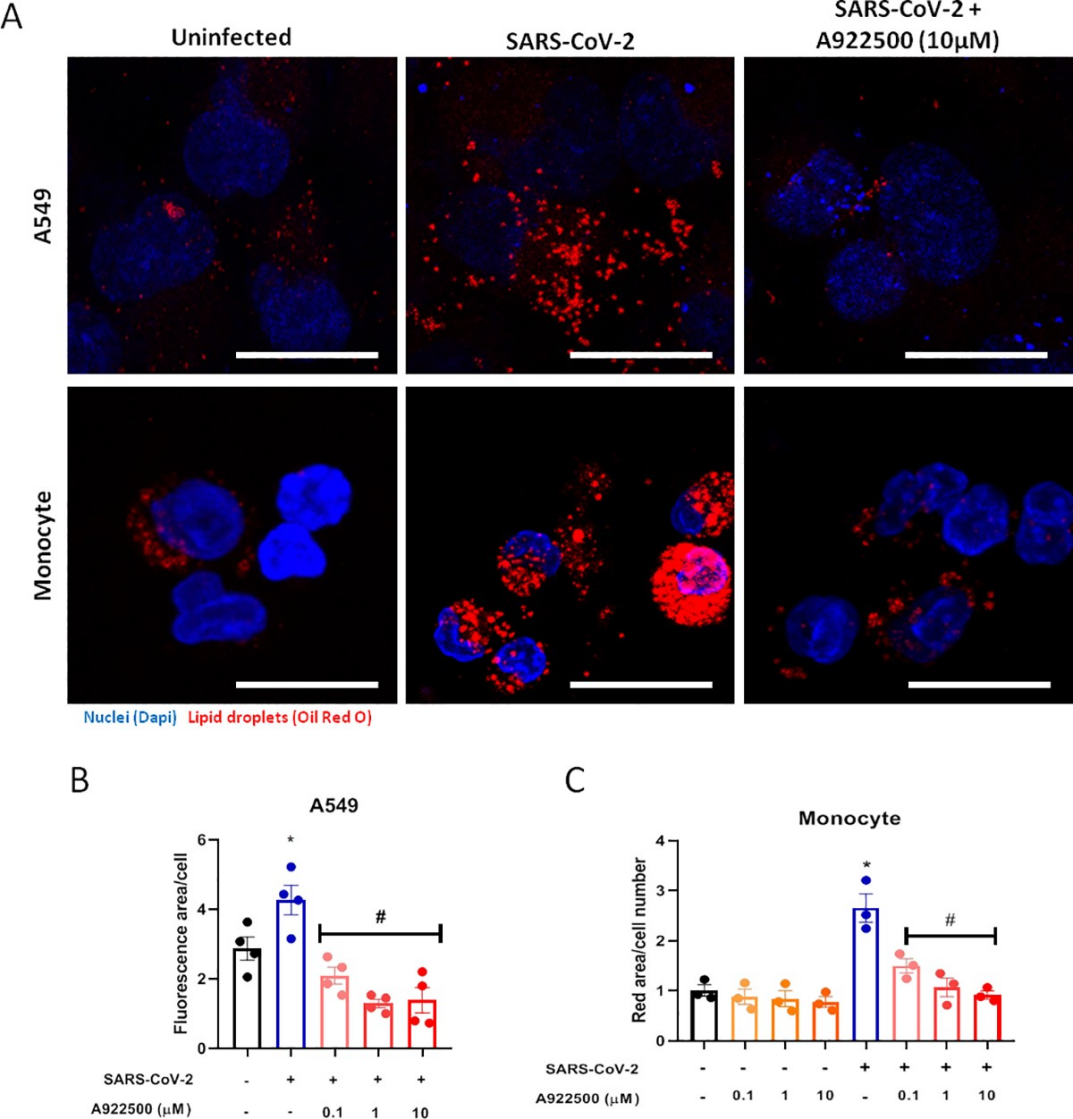
The A922500 inhibits lipid droplet biogenesis induced by SARS-CoV-2 in human pulmonary cells and monocytes. Human pulmonary cell (A549 cell line) and monocytes were pre-treated with DGAT-1 inhibitor A922500 for 2 hours before the infection with SARS-CoV-2 at MOI of 0.01 during 24h in monocytes and 48h in A549 cell line in the presence of the inhibitor. (A) LDs were captured by fluorescent microscopy after Oil Red O staining (Red) and nuclei stained with DAPI (Blue). Scale bar 20μm. (B and C) LDs were evaluated by ImageJ software analysis by the measurement of the fluorescent area of LDs from (B) A549 cells and (C) human monocytes pre-treated with A922500 using different concentrations (0.1, 1 and 10μM). Data are expressed as mean ± SEM obtained in four independent experiments and three independent donors. *p < 0.05 *versus* uninfected cells and #p < 0.05 *versus* infected cells.

DGAT-1 is a key enzyme involved in the final step of triacylglycerol synthesis and thus is central to remodel and finish the biogenesis of LDs [31]. During HCV infection, DGAT-1 was shown to be required for LD biogenesis, and to control HCV protein trafficking to LDs [32]. Consequently, DGAT-1 inhibition blocks HCV use of LDs as replication platforms and inhibits viral particle formation [21,32]. To assess the involvement of DGAT-1 in LD biogenesis during the SARS-CoV-2 infection, we treated Vero E6 and A549 cells at different concentrations of the A922500, an inhibitor of the enzyme DGAT-1, and evaluated the cell viability after 48 hours of the treatment. The values for 50% cytotoxic concentration (CC_50_) for the Vero E6 were 62.4 μM and the A549 cells were 58.4 μM (S2A and S2B Fig). Therefore, we treated the A549 and monocytes with A922500 for 2 hours at different concentrations prior to SARS-CoV-2 infection and the treatment was maintained until we evaluated the LD biogenesis 48 hours and 24 hours after infection, respectively. As shown by the representative images (Fig 2A) and quantification (Fig 2B and 2C), treatment with A922500 inhibited the LD formation triggered by SARS-CoV-2 infection in A549 human epithelial cells and in primary human monocytes in a dose dependent manner, with 50% effective concentration (EC_50_) value of 0.108 μM for A549 cells and 0.711 μM for human monocytes.

**Fig 3:**
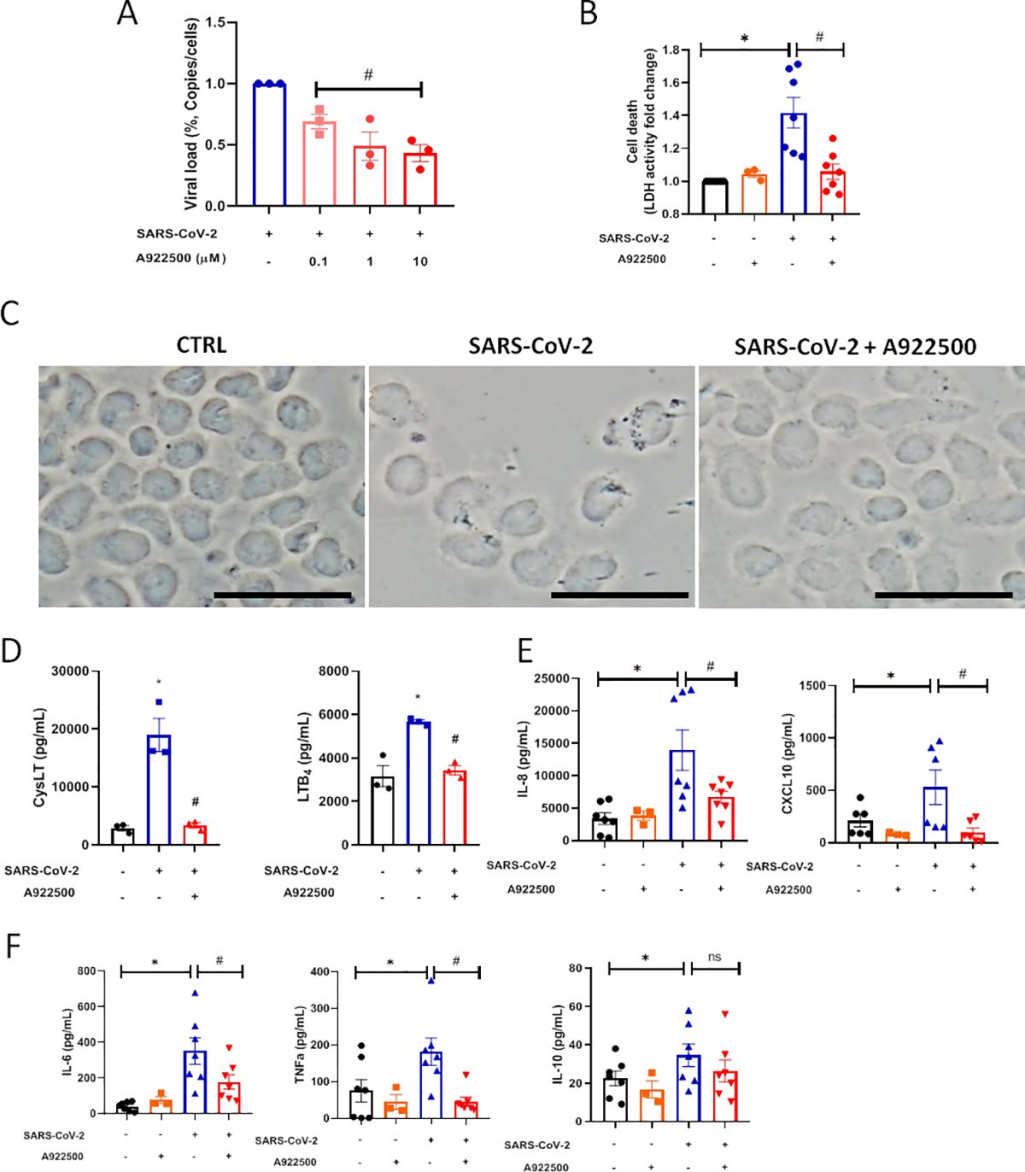
DGAT-1 Inhibitor A922500 decreases the pro-inflammatory profile and cell death induced by SARS-CoV-2 infection and reduces the viral load in human monocyte. Monocytes were pre-treated with DGAT-1 inhibitor A922500 in different concentrations (0.1, 1 and 10μM) for 2 hours before the infection with SARS-CoV-2 with MOI of 0.01 during 24h in presence of the inhibitor. (A) Cell death was measured in the supernatant by LDH activity fold change in relation to the uninfected cell. (B) Viral load by qPCR. Monocytes of each sample were counted for normalization. (C) Images of phase contrast from monocytes. Scale bar 20μm. (D-F) The inflammatory cytokines were measured in supernatants by ELISA (D) leukotrienes: CysLT and LTB_4_, (E) chemokines: IL-8 and CXCL10, (F) inflammatory cytokines: IL-6, TNF-α and IL-10. Data are expressed as mean ± SEM obtained in three independent donors for viral replication, leukotrienes and the group A922500 alone, and seven independent donors for the other groups. * p <0.05 *versus* uninfected cells and #p <0.05 *versus* infected cells.

Human monocytes infected with SARS-CoV-2 were shown to sustain viral genome replication, express higher levels of pro-inflammatory cytokines and may undergo cell death [33]. To gain insights on the functions of LDs in SARS-CoV-2 infection, LD biogenesis was inhibited by A922500, a DGAT-1 inhibitor. Treatment with A922500 significantly reduced the viral load in human primary monocytes in a dose dependent way (Fig 3A), suggesting a role for DGAT-1 and LD in SARS-CoV-2 replication.

It has already been demonstrated the capacity of the SARS-CoV-2 infection to induce cell death in monocytes, as evidenced by the leak of LDH to the extracellular space [34]. Cell death after viral infections can occur due to changes in the homeostasis of cellular compounds caused by the virus replication per se and/or by the heightened inflammatory response. Here, we measured the death of human primary monocytes infected with SARS-CoV-2 by the release of LDH into the supernatant, and also by the analysis of cell morphology observed in phase contrast. Our data show that SARS-CoV-2 triggered increased LDH release in the supernatant and that infected cells exhibited morphologic alterations with membrane rupture/damage compatible with necrosis (Fig 3B and 3C). The A922500 treatment alone did not alter the LDH release in uninfected cells supernatant. Similar to the observed for viral replication, DGAT-1 and LD inhibition with 10 μM of A922500 was able to inhibit SARS-CoV-2-induced cell death (Fig 3B and 3C).

### Lipid droplets are involved in SARS-CoV-2 heightened inflammatory response

Dysregulated immune response, with increased pro-inflammatory cytokine/chemokine production is observed during severe COVID-19 and associates with the outcome of the disease [35]. We observed that primary human monocytes infected with SARS-CoV-2 exhibit increased production of leukotrienes (LTB_4_ and cysLT), chemokines (IL-8 and CXCL10), inflammatory cytokines (IL-6, TNFα and IL-10) in comparison with uninfected cells (Fig 3D, 3E and 3F). Regarding other inflammatory mediators, SARS-CoV-2 infection increased IL-12 and reduced IL-4 production in comparison with uninfected monocytes.

LDs are organelles with major functions in inflammatory mediator production and innate signaling in immune cells [23,36,37]. To evaluate if LDs contribute to SARS-CoV-2-induced inflammation, monocytes were pre-treated with A922500, the secreted levels of lipid mediators, cytokine and chemokines were measured 24h after infection. It has been well established that LDs are organelles that compartmentalize the eicosanoid synthesis machinery and are sites for eicosanoid formation [37]. Here, we demonstrated that SARS-CoV-2 infection increased LTB_4_ and cysLT production in comparison with uninfected monocytes (Fig 3D). The treatment with DGAT-1 inhibitor A922500 reduced the synthesis of both lipid mediators by infected cells (Fig 3D). These data point out the importance of LD for the production of these pro-inflammatory lipid mediators. The A922500 treatment alone did not affect the inflammatory mediators production in uninfected cells (Fig 3E and 3F). We also observed that A922500 treatment downregulated the chemokines IL-8 and CXCL10, and the pro-inflammatory cytokines IL-6, TNFα (Fig 3E and 3F) and IL-12 (from 18.3 ± 3.2 in non-treated SARS-CoV2-infected cells to 6.08 ± 2.9 for A922500 treated and SARS-CoV2-infected; mean ± SEM), without affecting the anti-inflammatory cytokine IL-10 (Fig 3F). In addition to lowering the pro-inflammatory mediators, inhibition of LDs may shift the inflammatory profile by increasing the anti-inflammatory cytokine IL-4 (from 3.6 ± 1.3 in non-treated SARS-CoV-2-infected cells to 12.2 ± 1.7 for A922500 treated and SARS-CoV-2-infected; mean ± SEM).

Altogether, our data indicate that LDs have important functions in the modulation of inflammatory mediators production in SARS-CoV-2-infected monocytes and suggest that LD inhibition may reduce the dysregulated inflammatory process caused by SARS-CoV-2 infection.

### Lipid droplets are sites for SARS-CoV-2 replication

The up regulation of the lipid metabolism and LD biogenesis by the new SARS-CoV-2 suggest that the virus may explore host metabolism to favor it is replication using the LDs as a replication platform, as demonstrated for HCV [25,26,32], DENV [17] and rotavirus [22]. To evaluate this, we used a VERO E6 cell line that has a high replicative capacity for SARS-CoV-2.

**Fig 4:**
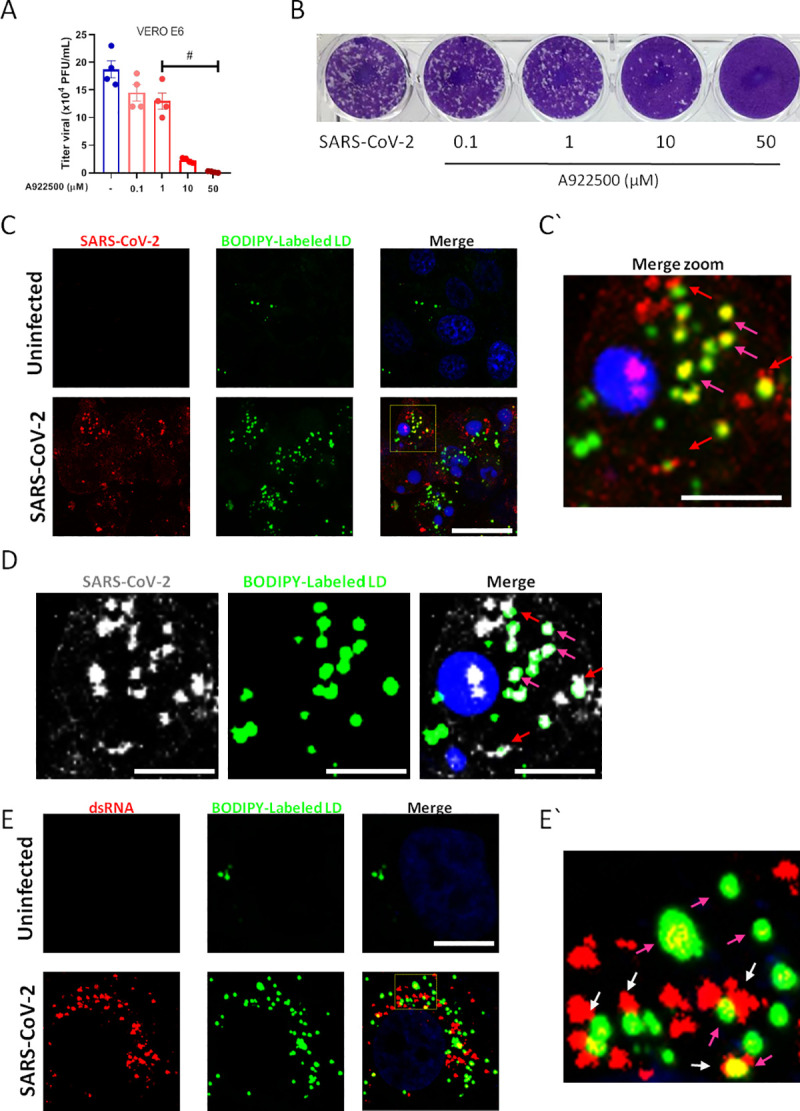
Lipid droplets are necessary for SARS-CoV-2 replication in VERO E6. VERO E6 were pre-treated with DGAT-1 inhibitor A922500 with different concentrations (0.1, 1, 10 and 50μM) for 2 hours before the infection with SARS-CoV-2 with MOI of 0.01 for 24h in presence of the inhibitor. (A) Viral replication was determined by Plaque assay. (B) Representative Plaque assay. (C-E) Immunofluorescence analyses of VERO E6 after SARS-CoV-2 infection with MOI of 0.01 for 48h. (C) The virus was detected by indirect immunofluorescence using convalescent donor serum (Red or white) or (E) the double strain RNA was detected by indirect immunofluorescence by J2 antibody (Red), the lipid droplets were stained with BODIPY 493/503 (Green) and nuclei stained with DAPI (Blue). (C’ and E’) Representative zoom images. Data are expressed of four independent experiments for SARS-CoV-2 replication and three for immunofluorescent analyses. #p <0.05 *versus* infected cells. Scale bar 20μm.

For these experiments, we pre-treated the VERO cells with a range of concentrations of DGAT-1 inhibitor A922500 (0.1–50 μM) for 2 hours, followed by infection with SARS-CoV-2 (MOI 0.01) for 24 hours in the presence of the inhibitor. The supernatant was used to measure the infectivity of progeny virus. Here, we observed that A922500 significantly inhibited SARS-CoV-2 replication in a dose dependent manner with 50% antiviral concentration (IC_50_) of 3.78 μM (Fig 4A and 4B).

To gain insights on the interaction of the SARS-CoV-2 with LDs we labeled the virus using immune serum from a convalescent COVID-19 patient that exhibit high anti-SARS-CoV-2 titers. For that, we stained the LDs using a BODIPY probe and analyzed the co-localization between the viral components and LDs by confocal microscopy. As shown in Fig 4C, intense immunoreactivity (red) was obtained in SARS-CoV-2 infected cells, whereas no labeling was observed in uninfected cells, indicative of specific SARS-CoV-2 labeling with COVID-19 convalescent serum (Fig 4C). As observed for monocytes and lung cells, Vero E6 infected cells increased LD biogenesis (green). Then, we examined the spatial relationship between SARS-CoV-2 and LDs. Confocal analysis showed a close apposition of SARS-CoV-2 immunoreactivity with BODIPY-labeled LDs (red arrows) and also co-localization of viral component(s) with BODIPY-labeled LDs (yellow; fuchsia arrows) in the infected cells (Fig 4C and 4D).

Increasing evidence points at host LDs play an important role in virus replicative cycle, including as hubs for viral genome replication and viral particle assembling [17,26,38,39]. To explore whether LDs are associated with SARS-CoV-2 replication, we use a specific antibody for double stranded (ds)-RNA (J2 clone). As shown in Fig 4E, we observed strong labeling of the ds-RNA in cells infected with the SARS-CoV-2 compared to uninfected cells. Similar to labeling detected with convalescent COVID-19 polyclonal serum, we observed close apposition and/or co-localization between BODIPY-labeled LDs and ds-RNA (Fig 4E and 4E`).

**Fig 5:**
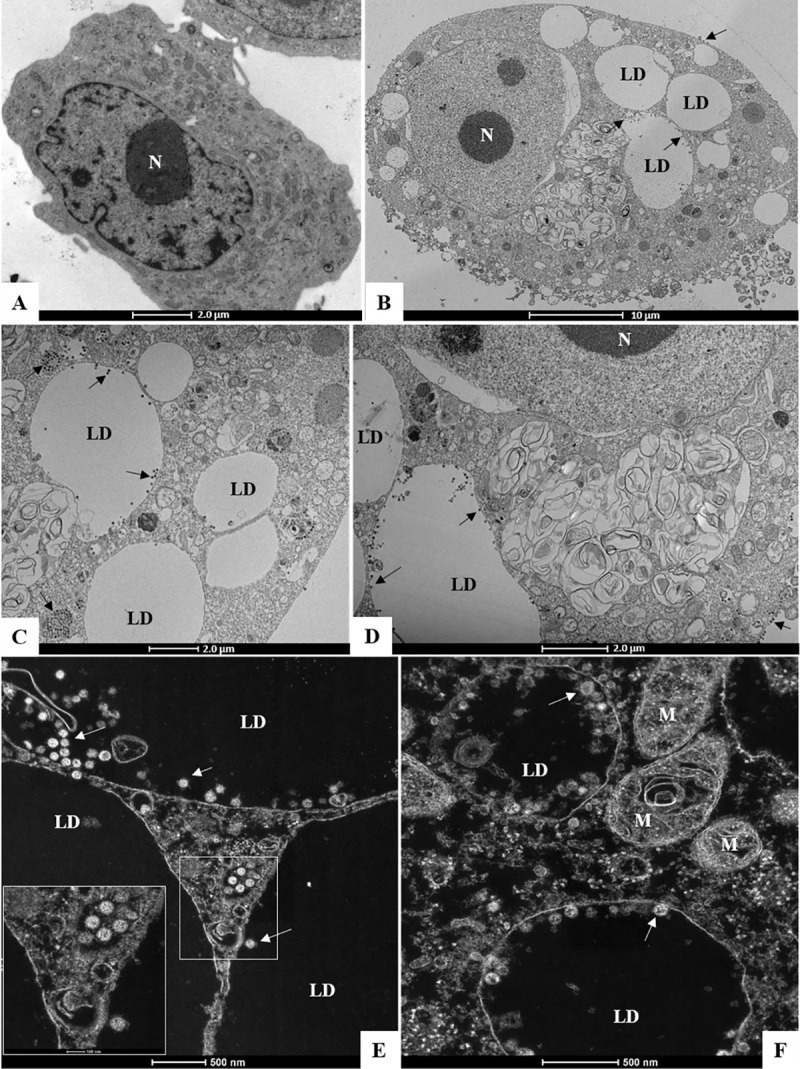
SARS-CoV-2 is associated with lipid droplet in Vero E6. Ultrastructural analyses by transmission electron microscopy of (A) uninfected Vero E6 Cell and (B-F) Vero E6 cells 48 hours post infection by SARS-CoV-2 (MOI 0.01). Arrows (virus particles), lipid droplet (LD), mitochondria (M), nucleus (N).

To gain further insights on LD SARS-CoV-2 interactions during virus cycle, Vero E6 infected with SARS-CoV-2 with MOI of 0.01 for 48h were analyzed by transmission electron microscopy. Confirming the previously shown LD induction in ORO stained cells, SARS-CoV-2 infection induced increased accumulation of LDs (Fig 5B) in comparison with uninfected cells (Fig 5A). Corroborating with the data obtained by confocal microscopy in Fig 4, we observed by transmission electron microscopy the viral particles in close association (arrows) with LDs (Fig 5C and 5D). Furthermore, we observed the presence of mounted viral particle within the LDs (Fig 5E and 5F). Although some viral particles can be visualized immersed in the LDs core of neutral lipid, most of the fully assembled viral particles are associated with the LD phospholipid monolayer. Moreover, the close position between LDs with ER and vesicles surrounded by lipid bilayer suggests cooperation of LDs with viral replication organelles for the intracellular traffic of viral particles, suggesting the LDs can actively participate as a replication platform for the SARS-CoV-2.

Collectively, our data suggest that SARS-CoV-2 uses LDs as a replication platform, and establish that pharmacological targeting of LD formation inhibit SARS-CoV-2 replication, emerging as a potential strategy for antiviral development.

## Discussion

Most positive-strand RNA virus are able to modulate the host lipid metabolism and to highjack LDs to enhance their fitness and replication/particle assembling capacity [23,24]. The pathways and mechanisms used may vary according to the virus and the host cell infected. The mechanisms and pathways explored by SARS-CoV-2 to support its replication within host cells are still largely unknown. Here we provide evidence that LDs participate in SARS-CoV-2 infection at two steps of host pathogen interaction: first, they are important players for virus replication; and second, they are central cell organelles in the amplification of inflammatory mediator production. First, we demonstrated that SARS-CoV-2 modulates pathways of lipid uptake and lipogenesis leading to increased LD accumulation in human host cells. We further showed that LDs are in close proximity with SARS-CoV-2 proteins and replicating genome, a finding suggestive that LDs are recruited as part of replication compartment. Second, we showed that inhibition of DGAT-1 blocked LD biogenesis, and reduced virus replication, cell-death and pro-inflammatory mediator production.

LD biogenesis is a multi-mediated and highly coordinated cellular process that requires new lipid synthesis and/or lipid uptake and remodeling, but the molecular mechanisms involved in LD formation during inflammation and infection are still not completely understood. Here, we showed the increased expression of SREBP-1 and the nuclear receptor PPARγ after SARS-CoV-2 infection indicative of reprogramming of cells towards a lipogenic phenotype. Accordingly, increased expression of SREBP-1 has been reported after respiratory viruses including MERS-CoV, SARS-CoV, and shown to regulate the increase of the LD and the accumulation of the cholesterol during the infection [40]. Consistently, targeting the SREBP-associated lipid biosynthetic pathways were shown to have antiviral properties [40]. The transcription factor PPARγ is activated by lipid ligands and promotes the expression of proteins involved in lipid homeostasis and LD biogenesis, and has been implicated in infectious and non-infectious LD biogenesis in monocytes/macrophages [41,42]. Based on these data we suggest that these two transcription factors may contribute to SARS-CoV-2 infection, favoring the lipid synthesis and LD formation. One important gene up regulated by PPARγ is the membrane receptor CD36 [43]. CD36 plays an important role in the transport and uptake of long-chain fatty acids into cells and participates in pathological processes, such as metabolic disorders and infections [44]. Previous reports showed that CD36 levels are increased in HCV and HIV-1 infections [45,46] and that it facilitates the viral attachment on host cell membrane contributing to viral replication [43]. Our results demonstrated that SARS-CoV-2 infection increases the CD36 expression in monocytes, suggesting that the increase of lipids uptake can contribute to LD formation, observed after the infection.

Numerous studies established LDs as key organelles during +RNA viruses replicative cycle [24]. Here, we observed strong labeling of the SARS-CoV-2 proteins and ds-RNA intimately associated to the LD and in some cases colocalizing with LD. Of note, the COVID-19 convalescent serum was used for viral protein immunolabeling and co-localization purpose. Convalescent serum, although provide specific labeling to the virus, might react to many viral antigens and positive labeling areas might contain different antigens. This may explain in part the lack of co-localization of some antigen signal with LDs. Further observation of viral particle association with LD was obtained by transmission electron microscopy adding support of a role of LDs in SARS-CoV-2 replication cycle. The other aspect that should be considered is that although LD may participate as a replication site, it is not exclusive and other cellular compartments may also have important roles in viral replication. Indeed, electron micrographs show LDs containing associated viral particles as well as LDs in close apposition with ER and virus containing vesicles suggesting that LDs may cooperate with other organelles for the intracellular traffic of viral particles. This fact highly suggests that SARS-CoV-2 recruits LDs to replication compartments and could use them as building blocks to fuel its own replication. Indeed, recent studies have shed light on active mechanisms of LD recruitment to viral replication compartments with bi-directional content exchange and essential functions to replication and virus particle assembly [26,39]. Future studies using viral protein specific antibodies would be necessary to better understand the mechanisms of interaction of SARS-CoV-2 with LDs. Our data support a role for LDs as assembly platform for viral replication.

DGAT-1, the key enzyme for triacylglycerol synthesis, is critical for LD biogenesis and mediates viral protein trafficking to LD by HCV and other viruses. Moreover, pharmacological suppression of DGAT-1 activity inhibits HCV replication at the assembly step [21,32]. We observed that DGAT-1 expression increases after SARS-CoV-2 infection and that this enzyme can contribute to the LD remodeling in the host cells. Pharmacological inhibitors of lipid metabolism protein are able to modulate the LD formation. Therefore, we used the DGAT-1 inhibitor (A922500) during SARS-CoV-2 infection and observed that this treatment reduced the LD biogenesis in monocytes and A549 cells, as well as decreased the viral load of SARS-CoV-2 in monocytes. Importantly, pharmacological suppression of DGAT-1 activity dose dependently inhibited SARS-CoV-2 infectious particle formation in Vero E6 cells with an IC_50_ of 3.78 μM. Thus, suggesting that DGAT-1 activity and LD formation are crucial to SARS-CoV-2 replication and assembly in these cells. Similar mechanisms involving DGAT-1 and LD have been reported to other viral infections [21,32]. Moreover, blockage of lipid metabolism and LD biogenesis by other compounds, such as TOFA, C75 and triacsin C also have been described to inhibit viral replication [22,47,48]. Future studies addressing different lipid metabolic pathways, including up-take and new synthesis, would be informative to understand the role played in SARS-CoV-2 replication cycle.

Dysregulated monocyte responses are pivotal in the uncontrolled production of cytokines during the infection with respiratory viruses, such as influenza A virus [49,50]. Dysregulated immune response with key involvement of monocytes, and increased pro-inflammatory cytokine/chemokine production is also observed during severe COVID-19 and is associated with the outcome of the disease [35,51]. SARS-CoV-2 infection of human monocytes in vitro recapitulates most of the pattern of inflammatory mediator production associated with COVID-19 severity, including the enhancement of the IL-6 and TNFα levels, and the consistent cell death, measured by LDH release [34,51,52]. We showed that SARS-CoV-2 infection generated a large amounts of mediators of inflammation by monocytes. Blockage of DGAT-1 activity lead to inhibition of the LDs accumulation and significantly reduced leukotriene production and pro-inflammatory cytokines released by monocytes, suggesting an important role for LDs to control the inflammatory process, and consequently to prevent the cell death-related with the uncontrolled inflammation. This finding is in agreement with the well-established role of LDs in inflammation and innate immunity [23,36,53]. Therefore, our data support a role for LD in the heightened inflammatory production triggered by SARS-CoV-2 and conversely, inhibition of LD biogenesis by targeting DGAT-1 activity may have beneficial effects on disease pathogenesis.

To our knowledge, this is the first report showing that LDs are increased in host cells and required for the formation of infectious virus particles during SARS-CoV-2 infection. In summary, our data demonstrate that SARS-CoV-2 triggers reprograming of lipid metabolism in monocytes and other cells leading to accumulation of LDs favoring virus replication. The inhibition of LD biogenesis modulates the viral replication and the pro-inflammatory mediator production. Therefore, our data support the hypothesis that SARS-CoV-2 infection increases the expression of the lipid metabolism-related proteins for their own benefit towards replication and fitness. Although, further studies are certainly necessary to better characterize the full mechanisms and importance of the LDs during the SARS-CoV-2 infection, our findings support major roles for LDs in SARS-CoV-2 replicative cycle and immune response. Moreover, the finding that the host lipid metabolism and LDs are required for SARS-CoV-2 replication suggests a potential strategy to interfere with SARS-CoV-2 replication by blocking the DGAT-1 and other lipid metabolic pathway enzymes.

## Methodology

### Ethics statement

Experimental procedures involving human monocytes from buffy coat from healthy donors were performed with samples obtained after written informed consent and were approved by the Institutional Review Board (IRB) of the Oswaldo Cruz Foundation/Fiocruz (Rio de Janeiro, RJ, Brazil) under the number 397–07. Experimental procedures involving human cells from COVID-19 patients and SARS-CoV-2 negative donors were performed with samples obtained after written informed consent from all participants or patients’ representatives according to the study protocol approved by the National Review Board (CONEP 30650420.4.1001.0008).

### Cells and reagents

Blood were obtained from reverse transcription polymerase chain reaction (RT-PCR) confirmed COVID-19 patients with 72h from intensive care unit in two reference centers (Instituto Estadual do Cérebro Paulo Niemeyer and Hospital Copa Star, Rio de Janeiro, Brazil) and SARS-CoV-2-negative health volunteers. Human monocytes were isolated from peripheral blood mononuclear cells (PBMCs) using density gradient centrifugation (Ficoll-Paque, GE Healthcare). The PBMC were resuspended in PBS containing 1 mM EDTA and 2% fetal bovine serum (FBS; GIBCO) to the concentration of 10^8^ cells/mL. The cells were incubated with anti-CD14 antibodies (1:10) for 10 min and magnetic beads-conjugates (1:20) for additional 10 min, followed by magnetic recovery of monocytes for 5 min. Recovered monocytes were resuspended in PBS containing 1 mM EDTA and 2% FBS and subjected to two more rounds of selection in the magnet according to the manufacturer’s instructions (Human CD14+ selection kit, Easy Sep; StemCell). The purity of monocyte preparations (>98% CD14+ cells) was confirmed through flow cytometry.

Human primary monocyte was obtained through plastic adherence of PBMCs. Briefly, PBMCs were isolated by Ficoll-Paque from peripheral blood or from buff-coat preparations of healthy donors. PBMCs (2 x 10^6^) were plated onto 48-well plates in low glucose Dulbecco’s modified Eagle’s medium (DMEM; GIBCO). After 2 hours of the plaque, non-adherent cells were washed out and the remaining monocytes were maintained for 24 hours in DMEM containing 5% inactivated male human AB serum (HS; Merck) and 100 U/mL penicillin-streptomycin (P/S; GIBCO) at 37°C in 5% CO_2_. The purity of human monocytes was above 90%, as analyzed by flow cytometry analysis (FACScan; Becton Dickinson) using anti-CD3 (BD Biosciences) and anti-CD16 (Southern Biotech) monoclonal antibodies.

Human lung epithelial carcinoma cell line (A549—ATCC/CCL-185) and African green monkey kidney (Vero subtype E6) were cultured in high glucose DMEM supplemented with 10% FBS and 100 U/mL P/S, and were incubated at 37°C in 5% CO_2_.

Human lung microvascular endothelial cell line (HMVEC-L—LONZA/CC-2527) was maintained following the manufacturer’s instructions. The cells were cultured in endothelial growth medium (EGM™-2MV BulletKit™, Clonetics) supplemented with 5% fetal bovine serum (FBS, Clonetics) and cells were incubated at 37°C and 5% CO_2_.

### Virus, infections and virus titration

SARS-CoV-2 was originally isolated from nasopharyngeal swabs of confirmed case from Rio de Janeiro/Brazil (GenBank accession no. MT710714). The virus was amplified in Vero E6 cells in high glucose DMEM supplemented with 2% FBS, incubated at 37°C in 5% CO_2_ during 2 to 4 days of infection. Virus titers were performed by the tissue culture infectious dose at 50% (TCID_50_/mL) and the virus stocks kept in -80°C freezers. According to WHO guidelines, all procedures involving virus culture were performed in biosafety level 3 (BSL3) multiuser facility. SARS-CoV-2 infections were performed at MOI of 0.01 in all cells with or without pre-treatment with the pharmacological inhibitor of DGAT-1 (A922500 –Sigma CAS 959122-11-3) for two hours and maintained after the infection. The Plaque-forming Assay was performed for virus titration in VERO E6 cells seeded in 24-well plates. Cell monolayers were infected with different dilutions of the supernatant containing the virus for 1h at 37°C. The cells were overlaid with high glucose DMEM containing 2% FBS and 2.4% carboxymethylcellulose. After 3 days, the cells were fixed with 10% formaldehyde in PBS for 3h. The cell monolayers were stained with 0.04% crystal violet in 20% ethanol for 1h. The viral titer was calculated from the count of plaques formed in the wells corresponding to each dilution and expressed as plaque forming unit per mL (PFU/mL).

### Cell viability assay

Vero E6 and A549 cells were seeded in 96-well plates. Then, the cells were treated with a range of concentrations of the A922500 during 48h. Next, the cells were fixed using 3.7% formaldehyde for 20 minutes. The cell monolayers were stained with 1% crystal violet in 20% ethanol for 10 minutes. The cells were washed with water and the crystal violet were extracted using methanol. The crystal violet was read in a spectrophotometer in the wavelength of 595nm.

### Lipid droplet staining

Human primary monocytes, A549 cell line, and HMVEC-L cell line were seeded in coverslips. The cells infected or not were fixed using 3.7% formaldehyde. In addition, after isolation, the monocytes from COVID-19 patients were fixed using 3.7% formaldehyde and adhered in coverslips through cytospin (500 x g for 5 min). The LDs were stained with 0.3% Oil Red O (diluted in 60% isopropanol) for 2 min at room temperature. The coverslips were mounted in slides using an antifade mounting medium (VECTASHIELD®). Nuclear recognition was based on DAPI staining (1 μg/mL) for 5 min. Fluorescence was analyzed by fluorescence microscopy with an 100x objective lens (Olympus, Tokyo, Japan). The numbers of LDs were automatically quantified by ImageJ software analysis from 15 aleatory fields.

### Immunofluorescence staining

VERO E6 cells were seeded in coverslips and after 48h were fixed using 3.7% formaldehyde. Cells were rinsed three times with PBS containing 0.1 M CaCl2 and 1 M MgCl2 (PBS/CM) and then permeabilized with 0.1% Triton X-100 plus 0.2% BSA in PBS/CM for 10 min (PBS/CM/TB). Cells were stained with convalescent serum from a patient to identify with COVID-19 at 1:500 dilution for overnight, followed by a human anti-IgG-Alexa 546 at 1:1000 dilution for 1 h. The ds-RNA was labeled by mouse monoclonal antibody J2 clone—Scicons [54] at 1:500 dilution for overnight, followed by a mouse anti-IgG-Dylight 550 at 1:1000 dilution for 1h. LDs were stained with BODIPY493/503 dye (dilution 1:5000 in water) for 5 min. The coverslips were mounted in slides using an antifade mounting medium (VECTASHIELD®). Nuclear recognition was based on DAPI staining (1 μg/mL) for 5 min. Fluorescence was analyzed by fluorescence microscopy with an 100x objective lens (Olympus, Tokyo, Japan) or Confocal Microscopy (Laser scanning microscopy LSM710 Meta, Zeiss).

### SDS-PAGE and Western blot

After 24h of SARS-CoV-2 infection, monocytes were harvested using ice-cold lysis buffer pH 8.0 (1% Triton X-100, 2% SDS, 150 mM NaCl, 10 mM HEPES, 2 mM EDTA containing protease inhibitor cocktail—Roche). Cell lysates were heated at 100°C for 5 min in the presence of Laemmli buffer pH 6.8 (20% β-mercaptoethanol; 370 mM Tris base; 160 μM bromophenol blue; 6% glycerol; 16% SDS). Twenty μg of protein/sample were resolved by electrophoresis on SDS-containing 10% polyacrylamide gel (SDS-PAGE). After electrophoresis, the separated proteins were transferred to nitrocellulose membranes and incubated in blocking buffer (5% nonfat milk, 50 mM Tris-HCl, 150 mM NaCl, and 0.1% Tween 20). Membranes were probed overnight with the following antibodies: anti-PPARγ (Santa Cruz Biotechnology, #SC-7196—H100), anti-CD36 (Proteintech-18836-1-AP), anti-SREBP-1 (Ab-28481), anti-DGAT-1 (Santa Cruz Biotechnology, #SC-271934) and anti-β-actin (Sigma, #A1978). After the washing steps, they were incubated with IRDye—LICOR or HRP-conjugated secondary antibodies. All antibodies were diluted in blocking buffer. The detections were performed by Supersignal Chemiluminescence (GE Healthcare) or by fluorescence imaging using the Odyssey system. The densitometries were analyzed using the Image Studio Lite Ver 5.2 software.

### Measurement of viral RNA load

Supernatants from monocytes were harvested after 24h of SARS-CoV-2 infection and the viral RNA quantified through RT-PCR. According to manufacter’s protocols, the total RNA from each sample was extracted using QIAamp Viral RNA (Qiagen®). Quantitative RT-PCR was performed using QuantiTect Probe RT-PCR Kit (Quiagen®) in a StepOne™ Real-Time PCR System (Thermo Fisher Scientific). Amplifications were carried out containing 2× reaction mix buffer, 50 μM of each primer, 10 μM of probe, and 5 μL of RNA template in 15 μL reaction mixtures. Primers, probes, and cycling conditions recommended by the Centers for Disease Control and Prevention (CDC) protocol were used to detect the SARS-CoV-2 [55]. For virus quantification it was employed the standard curve method [56]. Cells of each sample were counted before the PCR analyses for normalization. The Ct values for this target were compared to those obtained to different cell amounts, 10^7^ to 10^2^, for calibration.

### Measurements of inflammatory mediators and LDH activity

The monocyte supernatant was obtained after 24 hours of SARS-CoV-2 infection with or without treatment with A922500 (10 μM). Cytokines and chemokines were measured in the supernatant by ELISA following the manufacturer's instructions (Duo set, R&D). LTB_4_ and cysLT were measured in the supernatant by EIA following the manufacturer's instructions (Cayman Chemicals). Cell death was determined according to the activity of lactate dehydrogenase (LDH) in the culture supernatants using a CytoTox® Kit according to the manufacturer’s instructions (Promega, USA).

### Transmission electron microscopy

Infected monolayers were trypsinized at 48h p.i. Cell suspensions were fixed in 2.5% glutaraldehyde in sodium cacodilate buffer (0.2 M, pH 7.2), post-fixed in 1% buffered osmium tetroxide, dehydrated in acetone, embedded in epoxy resin and polymerized at 60°C over the course of three days [57,58]. Ultrathin sections (50–70 nm) were obtained from the resin blocks. The sections were picked up using copper grids, stained with uranyl acetate and lead citrate [59], and observed using FEI Titan and Hitachi HT 7800 transmission electron microscopes.

### Statistical analysis

Data are expressed as mean ± standard error of the mean (SEM) at least of three and maximum of five independent healthy donors. The paired two-tailed *t*-test was used to evaluate the significance of the two groups. Multiple comparisons among three or more groups were performed by one-way ANOVA followed by Tukey’s multiple comparison test. p values < 0.05 were considered statistically significant when compared SARS-CoV-2 infection to the uninfected control (*) group or SARS-CoV-2 infection with A922500 treat group (#).

## Supporting information

S1 Fig SARS-CoV-2 induces an increase of the LD biogenesis in different human pulmonary cell lines. Human pulmonary cell lines were infected with SARS-CoV-2 at MOI of 0.01 for 48h. (A and C) LDs were captured by fluorescent microscopy after Oil Red O staining (Red) and nuclei stained with DAPI (Blue). (B and D) LDs were evaluated by ImageJ software analysis by the measurement of the fluorescent area. Data are expressed of three independent experiments. *p <0.05 versus uninfected cells. Scale bar 20μm.
(TIF)

S2 Fig Cell cytotoxicity after the A922500 treatment. Vero E6 and A549 were treated with a range of concentrations of the A922500 for 48h. (A and B) Cell viability using crystal violet staining of uninfected VERO E6 and A549 cells treated with A922500. Data are expressed as mean ± SEM obtained in four independent experiments.
(TIF)
