# Supplementary figures and images for: Lipid droplets fuel SARS-CoV-2 replication and production of inflammatory mediators
Source: PLoS Pathog. 2020 Dec 16;16(12):e1009127. doi: 10.1371/journal.ppat.1009127 (PMC7773323; doi:10.1371/journal.ppat.1009127)

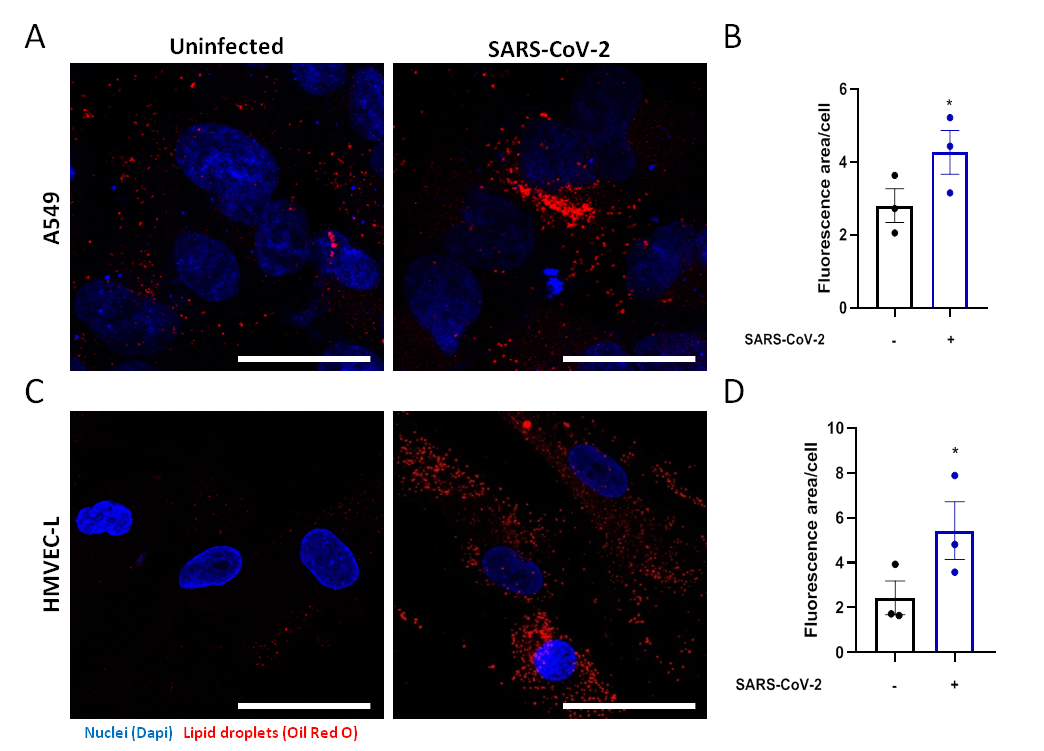

Supplement: S1 Fig — Human pulmonary cell lines were infected with SARS-CoV-2 at MOI of 0.01 for 48h. (A and C) LDs were captured by fluorescent microscopy after Oil Red O staining (Red) and nuclei stained with DAPI (Blue). (B and D) LDs were evaluated by ImageJ software analysis by the measurement of the fluorescent area. Data are expressed of three independent experiments. *p <0.05 versus uninfected cells. Scale bar 20μm. (TIF) [file ppat.1009127.s001.tif]

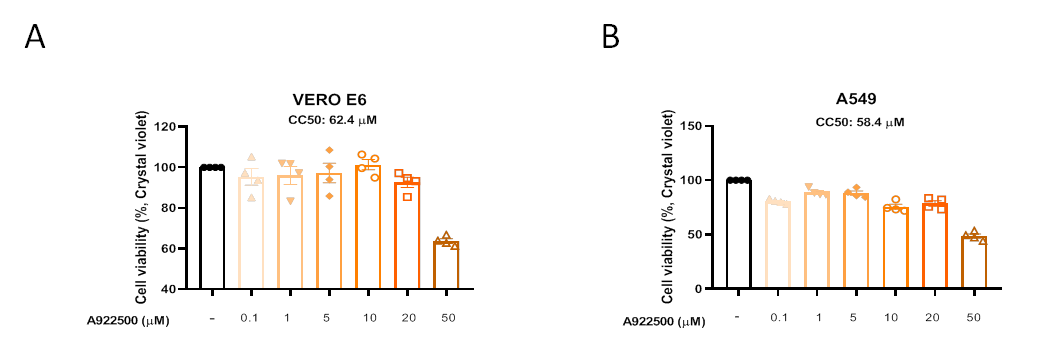

Supplement: S2 Fig — Vero E6 and A549 were treated with a range of concentrations of the A922500 for 48h. (A and B) Cell viability using crystal violet staining of uninfected VERO E6 and A549 cells treated with A922500. Data are expressed as mean ± SEM obtained in four independent experiments. (TIF) [file ppat.1009127.s002.tif]
