# Supplementary material for: Lipid droplets fuel SARS-CoV-2 replication and production of inflammatory mediators
Source: PLoS Pathog. 2020 Dec 16;16(12):e1009127. doi: 10.1371/journal.ppat.1009127 (PMC7773323; doi:10.1371/journal.ppat.1009127)
Supplement: Attachment — Submitted filename: Reply to reviewers.docx [file ppat.1009127.s003.docx]

**Reviewer #1 by** Dr. Ulrich Desselberger

***General Comments***

*In this manuscript evidence is presented that SARS-CoV-2 stimulates and interacts with lipid droplets (LDs) in infected human monocytes and different cell lines (A549, HMVEC-L, Vero E6). It is shown that in vitro infection of cells with SARS-CoV-2 reproduces the stimulation of LDs seen in ex vivo infected cells. Furthermore, chemical inhibition of a key enzyme of LD formation, diacylglycerol O-acyltransferase (DGAT-1), by compound A922500 inhibits the production of infectious progeny in SARS-CoV-2-infected cells. Modulation of pathways of lipogenesis in SARS-CoV-2-infected cells is observed. There is some co-localisation of viral proteins and the viral replicative dsRNA form with LDs. The pro-inflammatory innate immune response triggered by SARS-CoV-2 infection is reduced in A922500-pretreated, infected cells.*

*The data are of general interest, but deal with intracellular fatty acid biosynthesis and LD biogenesis in an incomplete way. The lack of uninfected, A922500-treated cells in Figs. 2 and 3 and their analyses is a major weakness. It may well be that the concentrations used of A922500 were non-toxic, but the evidence has to be presented in order to make the main data more convincing. The description of co-localisation data of viral proteins and viral replicative dsRNA with LDs should be rephrased.*

Thank you for your comments and suggestions. We have now added the controls of uninfected, A922500-treated cells in Figs. 2 and 3. As shown in the new figures, treatment with A922500 at the conditions used were non-toxic to the cells. As suggested, the description of co-localization data of viral proteins and viral replicative dsRNA with LDs were rephrased. In addition, we also included additional data by transmission electron microscopy that further give support to the claim of viral particles association with LD.

***Specific Comments***

*Line*

*58        Consider citation of: Andersen KG, Rambaut A, Lipkin WI, Holmes EC, Garry RF. The proximal origin of SARS-CoV-2. Nat Med. 2020;26(4):450-452. doi:10.1038/s41591-020-0820-9*

Added.

*74        Consider citation of: Chatel-Chaix L, Bartenschlager R. Dengue virus- and hepatitis C virus-induced replication and assembly compartments: the enemy inside--caught in the web. J Virol. 2014;****88****(11):5907-5911.*

Added.

*78        Consider citation of: Cheung et al, 2010 (in ref list) and: Gaunt ER, Cheung W, Richards JE, Lever A, Desselberger U. Inhibition of rotavirus replication by downregulation of fatty acid synthesis. J Gen Virol. 2013a;94(Pt 6):1310-1317; Gaunt ER, Zhang Q, Cheung W, Wakelam MJO, Lever AML, Desselberger U. Lipidome analysis of rotavirus-infected cells confirms the close interaction of lipid droplets with viroplasms. J Gen Virol. 2013b;94(Pt 7):1576-1586.*

Added.

*85        Blockage of intracellular fatty acid (FA) biogenesis (and LD formation) by other compounds, such as TOFA, C75 and triacsin C, and its influence on viral replication  have been described [Crawford SE, Desselberger U. Lipid droplets form complexes with viroplasms and are crucial for rotavirus replication. Curr Opin Virol. 2016;19:11-15; Lever A, Desselberger U. Rotavirus replication and the role of cellular lipid droplets: New therapeutic targets?. J Formos Med Assoc. 2016;115(6):389-394 (Table 1); Gaunt et al, 2013a, cited above]*

Thank you for the observation, citations and observations included.

*96        For HCV consider citation of Lee et al, 2019 (in ref. list).*

Done

*97        A reference relevant for noroviruses could be added: Doerflinger SY, Cortese M, Romero-Brey I, et al. Membrane alterations induced by nonstructural proteins of human norovirus. PLoS Pathog. 2017;****13****(10):e1006705.*

Included.

*102      … in vitro infection of cells and cell lines…*

Done

*104      Suppl. Fig. S1. In panel A, a photograph of the uninfected cell A549 is missing.*

Added.

*126      A reason should be given why cells were treated with A922500 for only 2 hours before viral infection. The highest non-toxic and lowest effective doses of the compound have to be determined in the experimental system used.*

Thank you for your observation, and we are sorry that the treatment procedure was not clear. We have now clarified the treatment procedure in the material and methods section: “SARS-CoV-2 infections were performed at MOI of 0.01 in all cells with or without pre-treatment with the pharmacological inhibitor of DGAT-1 (A922500 – Sigma CAS 959122-11-3) for two hours and maintained after the infection.”

*128f     and Fig. 2. In figure and text, relevant data on uninfected cells treated with A922500 are missing. The dose of 10 uM appears to be very high (with inhibitory doses 50% for different DGAT-1 enzymes being in the nanomolar range), and cells were pretreated with the compound for only 2 h. A dose response curve of treating uninfected cells with A922500 is missing. From experience with other inhibitors of the FA biosynthesis cascade it is known that inhibition of fatty acid biosynthesis and  LD formation can be cytotoxic and that the chemotherapeutic index of inhibitory compounds is often low.*

*138f     and Fig. 3. See comment on Fig. 2 above.*

Thank you for your observation. We have now added the appropriated controls of uninfected, A922500-treated cells in Figs. 2, Fig. 3 and Supplementary Figure 2. As shown in the new figures, treatment with A922500 at the conditions used were non-toxic to the cells.

*188      to line 199. Consider rephrasing, since SARS-CoV-2 antibody was used as a tool of detection of viral components. It is not clear which components were visualized, since a polyclonal convalescent serum was used. Figs. 4C and 4E show an incomplete merger of LDs and viral protein staining and viral dsRNA replicative form, respectively. Figs. 4C’ and 4E’ are not sharp and can be omitted.*

We have modified the text as suggested. Better quality images were added. In addition a new figure 5 containing transmission electron microscopy images of SARS-CoV2 infected Vero Cells were included adding support to the observations of viral particle association with LDs.

233      … lipogenic phenotype… Please clarify and provide a reference.

The text was modified for clarity and references added.

251      Was it considered to infect cells with SARS-CoV-2 in the presence of oleic acid in the medium?

We have not performed SARS-CoV-2 infection in the presence of oleic acid because oleic acid in addition to trigger LD formation may trigger different signaling pathways in monocytes that would lead to results difficult to interpret. Future studies using LD-specific proteins transfected cells would be informative but goes beyond the aim of the present study.

329      Fig. 1C uninfected is too dark.

Better quality image is now provided.

333      Fig. 1E. The intracellular FA biosynthesis pathway is only partially shown. See Fig. 1 of Crawford and Desselberger, 2016 [cited above].

Thank you for the observation. In Fig 1 we sought to highlight the branches of lipid metabolism pathways that were under analysis. This is now better explained in the text along with the observation that important branches would need to be further investigated in the future.

338      Fig. 1G. Consider phrasing: … Densitometric evaluation of data of panel 1F.

Thank you, rephrased.

342      Fig. 2. Data on uninfected cells treated with compound A922500 are missing. See comment above.

Data included, as mentioned above.

354      Fig. 3. See comment to Fig. 2 (line 342).

Data included, as mentioned above.

386      Fig. S1. See comment above.

Done

419      … endothelial cell line…

Done

426f     and 433f should be merged.

Done

469      and line 474. Indicate the pH of buffers.

Added

494      Consider citation of: Schmittgen, TD, Livak KJ. Analyzing Real-Time PCR Data by the Comparative CT Method. *Nat Prot* 2008*;* 3 (6): 1101–8.

 Added.

[827 words]

***Minor Comments***

*Line*

34        Consider reading: … biosynthetic needs. Thus, identifying…

Done

38        … lipid metabolism, energy homeostasis and intracellular transport…

Done

41        … were seen to modulate pathways of lipid synthesis and uptake as monitored by testing for CD36, SREBP-1, PPARgamma, and DGAT-1 expression … and triggered LD formation in different human cell lines…

Done

46        … mediators of pro-inflammatory response…

Done

64        … obligate intracellular parasites… [Not all viruses are pathogens.]

Corrected

68        … Increasing evidence points at… [see also line 200]

Corrected

76        … enzymes… reduce viral replication…

Done

85        … blocking LD biogenesis with a pharmacological inhibitor of … blocks viral replication… [Spell out DGAT-1 at first mentioning.

Done

87        … uncover details of viral manipulation…

Done

109      … insights into the mechanisms…

Done

116      Omit ‘and remodeling’.

129      … inhibited the LD formation triggered by… infection in a dose-dependent way…

Done

141      … changes in the homeostasis of cellular compounds…

182      … a high replicative capacity for SARS-CoV-2.

Ok

185      … the supernatant was used to measure the infectivity of progeny virus….

Modified

203      … To explore whether LDs are associated…

Modified

219      … participate in SARS-CoV-2 infection at two levels…

Modified

241      … Based on these data we suggest that these two transcription factors are important…

Done

249      … that … infection increases… suggesting that the increase…

Done

261      … is critical for LD biogenesis and mediates…

Done

262      and line 269. … pharmacological suppression of ..

Done

264      … can contribute to…

Done

268      … decreased the viral load…

Done

279      … SARS-CoV-2 infection … recapitulates…

Done

283      … mediators of inflammation…

Done

284      … inhibition of LD accumulation and…

Done

558      Ref. Codo et al, 2020 is incomplete.

Corrected

**Reviewer #2 by KJ Helbig, Ebony Alice Monson**

*The manuscript by da Silva Gomes Dias et al. describes the induction of lipid droplets in human monocytes taken from SARS-CoV-2 infected patients, as well as in cultured cell lines. It also demonstrates that inhibition of viral driven lipid droplet formation inhibits viral replication, as well as decreases cell death and the production of pro-inflammatory cytokines. This work offers further insight into the complex role of the lipid droplet in the context of viral infection, however, draws some definitive conclusions without sufficient evidence.*

Thank you for your observations. We have revised the text to tune down the conclusions as suggested.

*The induction of lipid droplets is a well shown phenomenon following many infectious diseases including bacteria and parasites, and there is evidence also demonstrating that activation of pattern recognition receptors can drive this response, suggesting it is a host driven response initially, rather than a pathogen driven response. However, the exact purpose of this event remains largely unknown. Additionally, it is well documented that multiple viruses alter the lipid dynamics in their host cells to*

*further their replication cycle, and it appears from the data presented in this manuscript, that it is likely that SARS-COV-2 also has a requirement for lipid in its viral life cycle given that inhibition of DGAT1, which is a key enzyme in the production of triacylglycerol synthesis, and underpins lipid droplet formation, reduced SARS-CoV-2 replication in vitro. Additionally, the data underpinning the statements that SARS-CoV-2 localises to and utilises lipid droplets as a replication platform, are not well supported by the limited imaging in the manuscript, and much work needs to be done to determine if indeed SARS-CoV-2 requires the lipid droplet as a platform for replication or assembly. Data investigating the longitudinal lipid droplet response may also assist in delineating the reliance of SARS-CoV-2 on lipid droplet’s as a replication platform, versus a ‘fuel’ source as is the case for flaviviruses.*

Thank you for your comments. We agree that lipid droplet induction is commonly observed during infections, including viral infections. Mechanisms of lipid droplet formation, composition and functions are highly regulated and may vary according to the cell and stimuli. To the best of our knowledge our study is the first to show lipid droplet formation and participation in SARS-CoV2 infection. Here we demonstrate that SARS-CoV-2 infection modulate cellular lipid metabolism in human cells favoring increased de novo lipid synthesis and lipid remodeling, leading to increased LD accumulation in different human cells. Moreover, our data on SARS-CoV2 association with LDs and the reduction of SARS-CoV-2 replication in vitro by inhibition of DGAT-1 are suggestive of a role for LDs in SARS-CoV2 cycle within host cells.

We have added electron microscopy analysis of SARS-CoV-2 infected Vero cells as to further support that SARS-CoV-2 localizes to LDs (new Figure 5).

Although we certainly agree that there are still numerous important open questions to understand the contributions of host lipid droplets to viral infections in general, and SARS-CoV2 in particular, we believe our data open an important avenue for investigation. Further studies will be necessary to establish the precise functions of LDs in SARS-CoV2 infection and the molecular mechanisms of SARS-CoV2 interaction with LDs and we have modified the discussion to reflect that.

*This manuscript also demonstrates that SARS-CoV-2 replication in monocytes upregulates many inflammatory cytokines, and that inhibition of lipid droplet formation with the use of DGAT1 inhibitors was able to reduce this increase and lessen cell death in vitro. However, the experiments are not well controlled, with no data presented for use of the DGAT1 inhibitor alone, in the context of no infection. Additionally, caution needs to be observed when interpreting the data, not only due to the lack of all controls, but also in light of the fact that the DGAT1 inhibitor also lessens viral replication, making it difficult to determine if it is the reduction in lipid droplets that results in the lowered inflammatory cytokine levels, or the reduction in viral replication; especially as replication data is not given for these experiments. This study is well written and integrative of the literature in the field. It represents numerous interesting findings in the context of SARS-CoV-2 and the ability of lipid droplets to affect the replication of the virus, and the production of inflammatory mediators. It will serve as a good preliminary set of data to underpin more extensive studies to truly delineate the role of these organelles, and DGAT1 in the life cycle of SARS-CoV-2.*

Thank you for your observation. We have now added the appropriated controls of uninfected, A922500-treated cells in Figs. 2, Fig. 3 and Supplementary Figure 2. As shown in the new figures, treatment with A922500 at the conditions used were non-toxic to the cells. Inhibition of viral load in monocytes mesuared by RT-PCR are shown in Figure 3 A.

Our data clearly show an effect of DGAT-1 inhibition in decrease lipid mediator production (LTB_4_ and cysLT) as well pro-inflammatory cytokines and chemokines after SARS-CoV2 infection. Whether the proinflammatory cytokine reduction induced by DGAT1 inhibition occurs through a direct effect on LDs or through indirect mechanisms are now discussed at discussion section.

**Reviewer #3 by** Dr. Prasert Auewarakul

*The manuscript by Dias et al shows an increased in lipid droplets in monocytes from COVID-19 patients. The same phenomenon was also observed in human monocytes and other cell lines infected with SARS-CoV2 in vitro. This increase in lipid droplets is probably explained by increases in expression of a number of genes involved in lipid uptake and synthesis. It was hypothesized that the virus induces these changes to promote viral replication and that lipid droplets play an important in viral replication. Applying an inhibitor of fatty acid synthesis could reduce the number of lipid*

*droplets in infected cell and the viral output as well as production of proinflammatory cytokines. The authors also tried to show co-localization between lipid droplets and viral antigens / dsRNA in infected cells. Except for the co-localization, the data seem to be reliable and trustworthy and in agreement with previous publications on a number of other viruses, which induced lipid synthesis and relied on the increased availability of intracellular lipid for optimal viral replication. While there are some colocalized areas between the viral antigens and lipid droplets, many areas of viral antigen do not colocalized with lipid droplet. This has to be better described and explained. Because the viral antigen detection was done using serum, which might react to many antigens and positive areas might contain different antigens. This may explain the lack of co-localization of some antigen signal. The colocalization with dsRNA looks less convincing as only a few co-localized dots were shown. The description and interpretation should be more cautiously stated.*

Thank you for your comment. We have expanded the description of the data as well as its current limitations at the discussion section. In addition, we have now provided new electron microscopy analysis of SARS-CoV2 infected Vero cells adding support to the observations of viral particle association with LDs. In addition, our confocal images of co-localizations and EM analysis are indicative that viral components/particles associate with LDs, but viral particles are not exclusively associated with LDs and this is now better clarified in the results and discussion section.

It is possible that inhibition of DGAT simply reduce the viral replication by reducing availability of intracellular lipid. Availability of lipid and number of lipid droplets is related but may not be the same thing.

DGAT-1 is a key enzyme involved in the final step of triacylglycerol synthesis and is central to the biogenesis of LDs. Specifically, DGAT incorporates exogenous FAs into TAGs. Importantly, DGAT1 functions in re-esterifying DAGs and MAGs that arise from hydrolysis of pre-existing TAGs to form new TAGs and pack them into LDs. As such, availability of total lipids is not expected to be much modified by DGAT inhibition, although the lipid species and numbers of LDs would. Future studies addressing different lipid metabolic pathways, including up-take and new synthesis, would be informative to understand the role played in SARS-CoV2 replication cycle but goes beyond the aim of the current MS. We have now discussed this aspect in the discussion section.

In addition, more information should be given on the COVID-19 patients, whose blood monocytes are shown in Fig. 1. How many patients were the data derived from, and what were their clinical conditions? Was there any correlation between the levels of lipid droplets and clinical severity?

Patients with severe COVID-19 were included in the study. We have added detailed information at Material and Methods and Figure legends. The number of patients included in the study and the lack of inclusion of patients with mild form of disease for the present study does not allow us to draw conclusion on association with disease severity. It would be an excellent aspect to investigate in the future.
